# Meta-research on pragmatism of randomized trials: rationale and design of the PragMeta database

**DOI:** 10.1186/s13063-023-07474-y

**Published:** 2023-06-30

**Authors:** Julian Hirt, Perrine Janiaud, Pascal Düblin, Lars G. Hemkens

**Affiliations:** 1 Pragmatic Evidence Lab, Research Center for Clinical Neuroimmunology and Neuroscience Basel (RC2NB), University Hospital Basel and University of Basel, Spitalstrasse 2, Basel, CH-4031 Switzerland; 2 Department of Clinical Research, University Hospital Basel and University of Basel, Basel, Switzerland; 3 Department of Health, Institute of Nursing Science, Eastern Switzerland University of Applied Sciences, St.Gallen, Switzerland; 4 Meta-Research Innovation Center at Stanford (METRICS), Stanford University, Stanford, CA USA; 5 Meta-Research Innovation Center Berlin (METRIC-B), Berlin Institute of Health, Berlin, Germany

**Keywords:** Pragmatic clinical trial [MeSH], Real-world clinical trials, Naturalistic randomized clinical trial, Databases, Bibliographic [MeSH]

## Abstract

**Background:**

Pragmatic trials provide decision-oriented, real-world evidence that is highly applicable and generalizable. The interest in real-world evidence is fueled by the assumption that effects in the “real-world” are different to effects obtained under artificial, controlled, research conditions as often used for traditional explanatory trials. However, it is unknown which features of pragmatism, generalizability, and applicability would be responsible for such differences. There is a need to provide empirical evidence and promote meta-research to answer these fundamental questions on the pragmatism of randomized trials and real-world evidence. Here, we describe the rationale and design of the PragMeta database which pursues this goal (www.PragMeta.org).

**Methods:**

PragMeta is a non-commercial, open data platform and infrastructure to facilitate research on pragmatic trials. It collects and shares data from published randomized trials that either have a specific design feature or other characteristic related to pragmatism or they form clusters of trials addressing the same research question but having different aspects of pragmatism. This lays the foundation to determine the relationship of various features of pragmatism, generalizability, and applicability with intervention effects or other trial characteristics.

The database contains trial data actively collected for PragMeta but also allows to import and link existing datasets of trials collected for other purposes, forming a large-scale meta-database. PragMeta captures data on (1) trial and design characteristics (e.g., sample size, population, intervention/comparison, outcome, longitudinal structure, blinding), (2) effects estimates, and (3) various determinants of pragmatism (e.g., the use of routinely collected data) and ratings from established tools used to determine pragmatism (e.g., the PRagmatic–Explanatory Continuum Indicator Summary 2; PRECIS-2).

PragMeta is continuously provided online, inviting the meta-research community to collaborate, contribute, and/or use the database. As of April 2023, PragMeta contains data from > 700 trials, mostly with assessments on pragmatism.

**Conclusions:**

PragMeta will inform a better understanding of pragmatism and the generation and interpretation of real-world evidence.

## Background

Pragmatic RCTs have been proposed to merge the advantages of using real-world data and following routine care (maximizing external validity) with the scientific rigor of RCTs (maximizing internal validity) [1]. Their main purpose is to inform decision-making in routine care, which requires high applicability and generalizability. Non-pragmatic, so-called explanatory, trials aim primarily at explaining the underlying mechanisms of treatment effects than to directly inform health care decisions. Pragmatic trials, their design, and their assessment are getting increasing attention with the creation of tools and initiatives such as the GetReal Trial Tool by the GetReal Initiative [2, 3]), a living textbook of pragmatic clinical trials by the National Institutes of Health [4], and the PRagmatic–Explanatory Continuum Indicator Summary 2 (PRECIS-2) to measure the pragmatism of a trial [5]. However, many trials labeled as “pragmatic” have features that are not compatible with routine care (e.g., the use of placebo control or double blinding), and the label “pragmatic” does not guarantee pragmatism and applicability of results [6, 7]. Many trials have several pragmatic features, but few have all or even most of them, and so it remains crucial to have a more in-depth understanding of these factors to better determine the impact of pragmatism on the estimation of treatment effects and evidence-based decisions.

Part of the emphasis on “real-world evidence” is the underlying assumption that studies with high generalizability and applicability would provide different treatment effect estimates than other studies. There is often the assumption that trials with non-pragmatic features that are typically done under artificial “ideal” and controlled settings with highly selected patients show stronger treatment effects on the desired endpoints [8, 9]. It is often argued this may result from better adherence [8], but we recently showed that adherence-adjusted effects are often similar to other effects [10]. It is also possible that including fewer patients with comorbidities leads to smaller differences with regard to adverse effects and harms. However, some empirical evaluations indicate that the overall degree of pragmatism can influence treatment effects estimates in meta-analyses [11, 12] and may also increase between-study heterogeneity [11, 13], which can limit the usefulness of meta-analyses for health technology assessments (and reimbursement decisions) [14] or clinical guidelines (and clinical decisions) [15]. Overall, the impact of features of pragmatism, generalizability, and applicability on treatment effects is unknown, and there is no large-scale systematic empirical evidence on this issue.

Here, we describe the rationale and design of the PragMeta database. The PragMeta database is a non-commercial, open data platform and infrastructure which contains information on randomized trials with various degrees of pragmatic features and it is designed to facilitate research on the pragmatism of randomized trials. These research projects may analyze the characteristics of pragmatic trials, assess their pragmatism, and/or empirically evaluate the meta-epidemiology of trial characteristics related to pragmatism, generalizability, applicability, and their impact on treatment effect estimates.

## Methods

In this methodological outline, we focus on the data infrastructure, processes, and general methods that are applied to feed the PragMeta database. PragMeta is managed using Directus, a free and open-source collaborative app to set up databases [16] that has been applied in other meta-research projects also lead by us [17, 18]. A data scientist (PD) developed the data infrastructure and website (www.PragMeta.org).

### Eligibility criteria

PragMeta contains trials that are randomized (excluding trials described as “quasi-randomized” or “controlled before and after design”) and fulfill one of the following conditions: ratings from established tools used to determine pragmatism available (e.g., overall or per domain PRECIS-2 score) or presence of a key determinant of pragmatism (e.g., self-labeled as “pragmatic”).

There are no restrictions on publication type and year.

### Data sources and organizational structure of PragMeta

#### Modules

The trials in PragMeta come from various sources, provided in a modular fashion. Modules are constructed around a specific research question and may have trial data that are provided via linkage and import of existing trials not identified and collected for the purpose of PragMeta or actively identified and collected for PragMeta (details on both types are provided in the following chapter).

The modules fall into two major categories with possible overlap: (a) collections of randomized trials that all share a specific design feature or other characteristic related to pragmatism and (b) collections of systematically identified randomized trials that may share a common design feature or a common clinical question and have information available on features related to pragmatism. Examples for (a) may be a collection of randomized trials that are self-labeled as “pragmatic” or that use routinely collected health data and examples for (b) may be a collection of randomized trials in a systematic review that were assessed with a tool such as PRECIS-2 for pragmatism or randomized trials in a disease/population.

First modules to actively collect data for PragMeta are underway and the description of specific underlying methods is published on Open Science Framework (OSF) [19]. These modules (as of April 2023) are shown in Table 1. All present modules, except PragSurgery, have been designed by the PragMeta team and are influenced by the clinical topics and research interest of our team. Additional modules may be initiated by the PragMeta team and/or collaborators in the future.

**Table 1:** Title and topic of ongoing modules that feed the PragMeta database (as of April 2023)

**Actively identified and collected for PragMeta**:
**PragCOVID**: Overview of COVID-19 randomized trials self-labeled as pragmatic. It included 37 trials
**PragMS**: Overview of pragmatic trials in multiple sclerosis [20]. All multiple sclerosis randomized trials likely to be pragmatic have been identified systematically and were assessed using PRECIS-2 for pragmatism. It includes 48 trials
**PragQoL**: Impact of pragmatism on the assessment of patient-reported outcomes (pain, fatigue, and quality of life) compared with objective clinical outcomes [21]. Cochrane reviews which include a pragmatic labeled trial in meta-analyses assessing pain, fatigue or quality of life and an objective outcome have been systematically identified. It includes 52 trials from 9 clusters
**Linkage and import of existing trials not identified and collected for the purpose of PragMeta**:
**PragEpi**: Association of pragmatism with treatment effect estimates [22]. This project collects trials from systematic review and meta-analyses citing and assessing PRECIS-2. It includes 185 trials from 18 clusters
**PragSurgery**: Collection of PRECIS-2 assessment in surgery trials. This module is the first large actively shared data collection integrated in PragMeta

Except from PragSurgery, all modules have been designed by the PragMeta team

#### Cluster

For modules that refer to category (b), systematically identified trials that share a common clinical question form a cluster; hence, such a module is fed by multiple clusters. While not all the trials in a cluster may share a specific design feature or other characteristic related to pragmatism (e.g., are labeled as “pragmatic”), they all assess effects in the same population, intervention, comparator, and outcome, but with different aspects of pragmatism (from none to all) or differences in other features related to pragmatism. Such trials may be very non-pragmatic and are included in PragMeta to determine the relationship of features of pragmatism, generalizability, and applicability with intervention effects or other characteristics.

### Linkage and import of existing datasets of trials

Existing datasets of randomized trials with common features or assessing the same clinical question which report the ratings of pragmatism using PRECIS-2 or another tool per trial (see category b above) can be systematically imported into the PragMeta database. Currently, we have systematically identified all systematic reviews and meta-analyses that have cited and assessed the PRECIS-2 (see Table 1 module PragEpi). We are also importing datasets non-systematically identified and/or provided by collaborators and research partners.

### Active data collection: Index trial approach

To identify clusters of trials reflecting a broad spectrum of aspects of pragmatism, an “index trial approach” can be used. This approach allows an efficient collection of relevant trials with various degrees of pragmatism on the same clinical question. Since most trials conducted thus far are not pragmatic, this approach increases the chance that identified collections of trials answering the same research questions include at least one pragmatic trial and therefore reflect a broader spectrum of aspects of pragmatism. Index trials are such trials, presenting one or more features that indicate potential pragmatism (e.g., are labeled as “pragmatic”). They are used as starting point to obtain multiple other trials presenting variable aspects of pragmatism and addressing the same topics. Such trials are referred to as corresponding trials. It is assumed that trials that are all included in the same meta-analysis of a clinical systematic review address the same topic. This approach is efficient as it relies on available systematic searches from clinical systematic reviews (e.g., Cochrane reviews), ensuring a comprehensive set of multiple studies on the same research question.

#### Identification of index trials

A sample of index trials can be obtained by searches in literature databases using a search component focusing on pragmatic trials. Either a simplified search to identify RCTs self-labeled as “pragmatic” in title and/or abstracts (i.e., “(pragmatic$ or naturalistic) and trial).ab,ti.”) or a more complex search string designed for, e.g., Ovid MEDLINE may be used [23]. Depending on the module of interest, other relevant components may be added to the search strategy.

#### Identification of corresponding trials

To identify corresponding trials, automatic citation-based searches are used (e.g., with the iCiteR package for R [24]) to search for systematic reviews (e.g., Cochrane reviews) citing the index trial of interest. The focus is on Cochrane reviews due to their high methodological standards, and transparent and standardized reporting [25], but using other original systematic reviews is possible.

Once relevant citing systematic reviews are identified, it needs to be determined if the index trial is actually included with other trials on the same clinical question. For Cochrane reviews, this can be assumed if the index trial is meta-analytically combined with other trials, the procedure would then be as follows: (1) check if the index trials are listed in the section “References to studies included in this review” and if it is included in a meta-analysis. If multiple meta-analyses within a Cochrane review or across multiple reviews include the index trial, the selection of the eligible meta-analysis may be guided by the following hierarchy of rules:

- Meta-analysis reporting the outcome of interest of our module (if not defined, next level or if more than one, go to level d)
- Meta-analysis reporting the primary outcome of our index RCT (if not available or more than one, next level)
- Meta-analysis reporting the primary outcome as identified by the Cochrane review in which it is included (if does not include the identified index RCTs or more than one, next level)
- Meta-analysis reporting the largest number of RCTs (if more than one, next level)
- Meta-analysis reporting the largest total sample size

This process may be adapted for types of evidence synthesis other than Cochrane reviews.

### Data entry

PragMeta data may come from data import or active extraction as described above. The database feeding procedure is organized in six hierarchical collections: systematic reviews, meta-analyses (included in the systematic reviews), trials (included in the meta-analyses or individual), comparisons (in the trials), effects (of the comparisons), pragmatism assessments. Thus, to an entry of a superordinated collection (e.g., systematic reviews or meta-analyses), one or more entries of a subordinated collection (e.g., trials) may be assigned. For example, a systematic review may contain several meta-analyses, or a meta-analysis contains several trials, and, specifically, a trial may be assigned to multiple systematic reviews and/or meta-analyses.

The data source for extractions at the systematic reviews, meta-analyses, comparisons, and effects level is the systematic review (e.g., Cochrane review); for extractions at the trials and pragmatism assessments level, the data source is the original trial report.

**Fig. 1:**
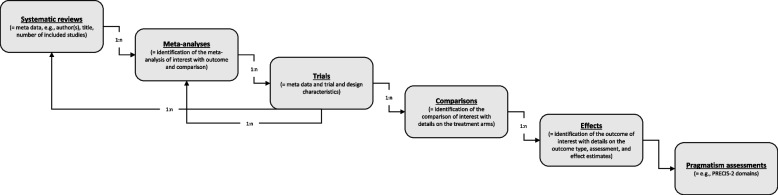
Structure of the PragMeta database used for data collection and hierarchical management of collections (figure design inspired by Ladanie et al. [26]). n, number; PRECIS-2, PRagmatic–Explanatory Continuum Indicator Summary 2

An overview of core variables per collection is given in Table 2. How the collections are related is shown in Fig. 1. A comprehensive list of variables and explanations can be found in the procedure document on OSF [19].

**Table 2:** Core variables per collection

Variable	Data type	Description
Systematic reviews		
Digital objective identifier (DOI)	Alphanumeric	Unique Digital Object Identifier
Publication year	Categorical	Year of publication
First author	Alphanumeric	Name of the first author
Title	Alphanumeric	Title of the publication
Number of studies included	Numeric	Number of studies included in the quantitative synthesis of the systematic review
Meta-analyses		
Number of trials included	Numeric	Number of trials included in the meta-analysis regardless if they are eligible for PragMeta or not
Comparison	Alphanumeric	Details on the comparison assessed in the meta-analysis, e.g., “Shared decision-making versus usual care”
Outcome	Alphanumeric	Outcome used in the meta-analysis
Trials		
DOI	Alphanumeric	Unique Digital Object Identifier
Publication year	Categorical	Year of publication
First author	Alphanumeric	Name of the first author
Title	Alphanumeric	Title of the publication
Trial category	Categorical	Index or corresponding trial
Trial registration	Alphanumeric	Any identification number that can be traced back to an official trial registry
Country of conduct	Alphanumeric	Country or countries of conduct
Trial purpose	Categorical	E.g., treatment, prevention, supportive care
Funding	Categorical	E.g., funded by industry/for profit or public/not-for-profit
Patient representatives	Categorical	Indicates if author mention the participation of patients’ representatives at any level of conceptualization, conduct, or reporting of the trial
Disease	Alphanumeric	Brief description of the disease
Therapeutic area	Categorical	E.g., cardiology, neurology, dermatology
Patient type	Categorical	Population or setting, e.g., outpatients, healthy volunteers, general population
Randomization	Categorical	Assessment of the randomization process
Blinding^a^	Categorical	None, single, double, or unclear
Longitudinal structure	Categorical	Parallel, crossover, factorial, or other
Advanced design features	Categorical	E.g., platform, adaptive, remote
Number of sites	Numeric	Number of trials sites/study centers
Number of arms	Numeric	Number of trial arms
Methods to collect informed consent^a^	Categorical	Waived, written, orally, in person, online, unclear, not reported (multiple choices)
Use of routinely collected data^a^	Categorical	Yes, no, unclear
Comparisons		
Intervention type	Categorical	E.g., care management, drug, lifestyle
Comparison type^a^	Categorical	E.g., care management, drug, lifestyle
Backbone	Categorical	If there is a common backbone therapy
Effects		
Outcome	Alphanumeric	Brief description of the outcome
Outcome type^a^	Categorical	E.g., clinical, surrogate, biomarker
Outcome reported by	Categorical	E.g., investigator, patient, carer
Outcome assessor blinded	Categorical	Yes, no, partly, unclear
Length of follow-up in months	Numeric	Number of months as the patient being followed during the trial until the latest measure of the outcomes
Metric	Alphanumeric	Metric of the estimated mean difference or of the estimated ratio, e.g., odds ratio, mean change
Dispersion variable	Alphanumeric	E.g., standard deviation, standard error
Outcome direction	Categorical	Whether the outcome occurs more or is improved in participants in the intervention arm compared with comparison arm or in participants in the comparison arm compared with intervention arm
Outcome scale	Categorical	Whether the outcome translate a positive (e.g., clinical improvement) or negative effect (e.g., death) for the participant
Participants in intervention arm	Numeric	Number of participants in the intervention arm
Participants in comparison arm	Numeric	Number of participants in the comparison arm
Events intervention arm	Numeric	Number of events in the intervention arm
Events comparison arm	Numeric	Number of events in the comparison arm
Estimated ratio	Numeric	Estimated ratio of intervention and comparison arm
Estimated ratio lower limit	Numeric	Estimated ratio lower limit of 95% confidence interval
Estimated ratio upper limit	Numeric	Estimated ratio upper limit of 95% confidence interval
Pragmatism assessment		
PRECIS-2 Domain		
Eligibility^b^	Alphanumeric	Who is/which clusters are selected to participate in the trial?
Recruitment^b^	Alphanumeric	How are participants/clusters recruited into the trial?
Setting	Alphanumeric	Where is the trial being done?
Organization	Alphanumeric	What experience and resources are needed to deliver the intervention?
Flexibility: delivery	Alphanumeric	How should the intervention be delivered?
Flexibility: adherence	Alphanumeric	What measures are in place to make sure participants adhere to the intervention?
Follow-up	Alphanumeric	How closely are participants followed-up?
Primary outcome	Alphanumeric	How relevant is it to participants?
Primary analysis	Alphanumeric	To what extent are all data included?

^a^Potential determinants of pragmatism

^b^In case of cluster-RCTs, this domain is assessed on the individual participant level and on the cluster level, respectively

The level of data entry may vary between specific modules and imported existing datasets, and thus missing data may occur. For imported existing datasets, datasets are matched to our database infrastructure, but no additional data quality controls are carried out. For modules conducted by us, one reviewer enters/extracts and assesses the included trials. This procedure is complemented through entry/extraction and assessment by a second reviewer (independently in duplicate or not, depending on the module). The backend infrastructure of the database enables to indicate the entry/extraction status as well as the possibility to keep track of verifications within each of the six collections. PRECIS-2 assessments done by the PragMeta team are performed by one reviewer or by two independent reviewers (depending on the module). The infrastructure of the database allows to compare multiple PRECIS-2 assessments and to designate a consented version that is shown on the PragMeta website. Furthermore, as indicators of data quality, the data source, and who extracted the data and/or rated pragmatism using PRECIS-2 or another tool (e.g., PragMeta team, publication team of the original dataset, trial investigators) is always recorded. The frequency for updating the search, screening, and data entry may vary between specific modules.

The PragMeta database is publicly available and can be downloaded. For contributions and collaborations including active data entry and data import, we invite research groups to contact us.

### Data on pragmatism

The information collected in PragMeta about pragmatism, for now, includes the assessment of PRECIS-2 which comprises 9 domains addressing features of a RCT that might impact the pragmatic-explanatory continuum (Table 2). Each domain receives a score based on a 5-point Likert scale (1 = very explanatory; 3 = equally pragmatic and explanatory; and 5 = very pragmatic; or no information). We also collect specific trial characteristics or determinants, not captured by PRECIS-2, that may be applicable to more pragmatic or explanatory approaches. Such determinants include, for example, (i) the use of routine collected data and how they are used (e.g., to recruit and/or to collect outcome data), (ii) how is informed consent collected, (iii) use of blinding (e.g., double-blind), (iv) type of control (e.g., placebo or standard of care), and (vi) type of outcome (e.g., patient-reported or surrogate outcome). PragMeta can be expanded by further variables, reflecting for example further determinants of pragmatism identified in due course.

### Data sharing

PragMeta is an open data platform. The cleaned datasets are continuously provided online and can be downloaded in various formats.

## Conclusions

The PragMeta database serves as a collection of trials that are used for multiple and diverse research projects on the generalizability, applicability, and pragmatism of clinical trials; including, but not limited to, meta-epidemiological studies on the comparison of treatment estimates between trials with different levels of pragmatic and explanatory features. PragMeta is, to our knowledge, the first freely available, large-scale, collaborative database with information on pragmatism, other design features, and treatment effects.

Overall, with the PragMeta database, we aim at providing a platform and an infrastructure for meta-research on pragmatic trials, and we invite other research groups to contribute to and/or use the PragMeta database. By following this collaborative idea to generate a large sample of pragmatic trials that assess health interventions, we aim to better understand the determinants, characteristics, and impact of pragmatic trial design features.

PragMeta aims to better understand and to quantify the relationship of trial pragmatism and treatment effects and to determine which trial features are key drivers for differences between treatment effects in more artificial research settings versus more practical real-world settings and hopefully result into a more standardized methodological framework that can be used by researchers, clinicians, and regulators and to better generate, use, and apply clinical research results.

## Data Availability

Not applicable.

## Abbreviations

OSF: Open Science Framework
PRECIS-2: PRagmatic Explanatory Continuum Indicator Summary tool
RCD: Routinely collected health data
RCT: Randomized clinical trial
